# Pyruvate dehydrogenase complex and lactate dehydrogenase are targets for therapy of acute liver failure

**DOI:** 10.1016/j.jhep.2018.03.016

**Published:** 2018-08

**Authors:** Rosa Ferriero, Edoardo Nusco, Rossella De Cegli, Annamaria Carissimo, Giuseppe Manco, Nicola Brunetti-Pierri

**Affiliations:** 1 Telethon Institute of Genetics and Medicine, Pozzuoli, Italy; 2 Institute for Applied Mathematics 'Mauro Picone', National Research Council, Naples, Italy; 3 Institute of Protein Biochemistry, National Research Council, Naples, Italy; 4 Department of Translational Medicine, Federico II University of Naples, Naples, Italy

**Keywords:** Pyruvate dehydrogenase complex, Lactate dehydrogenase, Acute liver failure, Galloflavin

## Abstract

**Background & Aims:**

Acute liver failure is a rapidly progressive deterioration of hepatic function resulting in high mortality and morbidity. Metabolic enzymes can translocate to the nucleus to regulate histone acetylation and gene expression.

**Methods:**

Levels and activities of pyruvate dehydrogenase complex (PDHC) and lactate dehydrogenase (LDH) were evaluated in nuclear fractions of livers of mice exposed to various hepatotoxins including CD95-antibody, α-amanitin, and acetaminophen. Whole-genome gene expression profiling by RNA-seq was performed in livers of mice with acute liver failure and analyzed by gene ontology enrichment analysis. Cell viability was evaluated in cell lines knocked-down for PDHA1 or LDH-A and in cells incubated with the LDH inhibitor galloflavin after treatment with CD95-antibody. We evaluated whether the histone acetyltransferase inhibitor garcinol or galloflavin could reduce liver damage in mice with acute liver failure.

**Results:**

Levels and activities of PDHC and LDH were increased in nuclear fractions of livers of mice with acute liver failure. The increase of nuclear PDHC and LDH was associated with increased concentrations of acetyl-CoA and lactate in nuclear fractions, and histone H3 hyper-acetylation. Gene expression in livers of mice with acute liver failure suggested that increased histone H3 acetylation induces the expression of genes related to damage response. Reduced histone acetylation by the histone acetyltransferase inhibitor garcinol decreased liver damage and improved survival in mice with acute liver failure. Knock-down of PDHC or LDH improved viability in cells exposed to a pro-apoptotic stimulus. Treatment with the LDH inhibitor galloflavin that was also found to inhibit PDHC, reduced hepatic necrosis, apoptosis, and expression of pro-inflammatory cytokines in mice with acute liver failure. Mice treated with galloflavin also showed a dose-response increase in survival.

**Conclusion:**

PDHC and LDH translocate to the nucleus, leading to increased nuclear concentrations of acetyl-CoA and lactate. This results in histone H3 hyper-acetylation and expression of damage response genes. Inhibition of PDHC and LDH reduces liver damage and improves survival in mice with acute liver failure. Thus, PDHC and LDH are targets for therapy of acute liver failure.

**Lay summary:**

Acute liver failure is a rapidly progressive deterioration of liver function resulting in high mortality. In experimental mouse models of acute liver failure, we found that two metabolic enzymes, namely pyruvate dehydrogenase complex and lactic dehydrogenase, translocate to the nucleus resulting in detrimental gene expression. Treatment with an inhibitor of these two enzymes was found to reduce liver damage and to improve survival.

## Highlights

• Pyruvate dehydrogenase complex (PDHC) and lactate dehydrogenase (LDH) translocate to the nucleus in injured liver cells.
• Increased nuclear PDHC and LDH induce increased nuclear acetyl-CoA and lactate, histone hyper-acetylation, and damage response gene expression.
• Inhibition of histone acetylation or inhibition of PDHC and LDH reduce damage and inflammation in injured livers.
• Galloflavin inhibits both PDH and LDH and protects from damage and mortality induced by hepatotoxins.

## Graphical abstract

**Figure:**
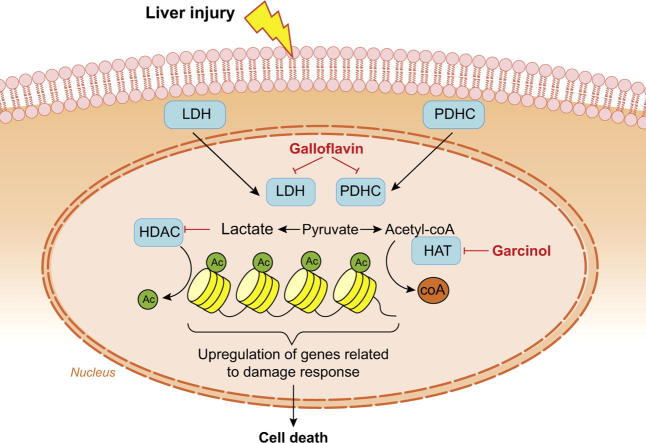

## Introduction

Acute liver failure is a rapidly progressive, life-threatening deterioration of liver function that results in altered neurological state and coagulopathy. The only treatment that improves the outcome of acute liver failure is emergency liver transplantation. Because liver transplantation has limitations related to invasiveness, limited donor availability and high mortality, therapies for acute liver failure are urgently needed.

Moonlighting of metabolic enzymes in the nucleus has been recognized as an important mechanism linking metabolic flux to regulation of gene expression.^1^ Dynamic nuclear translocation of pyruvate dehydrogenase complex (PDHC) and increased acetyl-CoA affect histone acetylation and epigenetic regulation.^2^ Lactate dehydrogenase (LDH) is also detected in the nucleus[3], [4] where it generates lactate. Lactate has global effects on gene transcription which overlap with the changes induced by histone deacetylase (HDAC) inhibitors.^5^

In this study, we found that levels and activities of both PDHC and LDH are increased in the nuclear fractions of livers of mice exposed to acute hepatic injuries. Their increase was associated with higher concentrations of acetyl-CoA and lactate in nuclear fractions, and marked increase in histone acetylation. In addition, we showed that pharmacologic inhibition of LDH and PDHC reduces liver injury and improves mouse survival.

## Materials and methods

### Mouse experiments

Mouse studies were approved by the Italian Ministry of Health. Mice were group-housed (2–5 mice per cage) in standard mouse cages and maintained under a 12 h light/dark cycle with free access to water and standard mouse chow. Wild-type six-week-old male C57BL/6N mice (weight: 20–22 g) purchased from Charles River Laboratories were injected intraperitoneally (i.p.) with 0.5 μg/g of body weight of CD95-activating antibody (CD95-Ab; BD Biosciences), 0.6 μg/g of body weight of α-amanitin (Sigma-Aldrich), 0.4 mg/g of body weight of acetaminophen (APAP) (Sigma-Aldrich), or vehicle (saline). 20 mg/kg/day of garcinol (Enzo Life Sciences) or vehicle (saline) was injected i.p. for five days prior to the injection of CD95-Ab. Galloflavin at the doses of 10, 20, 40 or 80 mg/kg diluted in 40% dimethyl sulfoxide (DMSO) or vehicle (40% DMSO) was injected i.p. at the same time as CD95-Ab, α-amanitin, or APAP injections and was continued daily until mouse sacrifice by cervical dislocation at 72 h after CD95-Ab, α-amanitin, or APAP injections. Blood samples were collected by retro-orbital bleedings and were analyzed for alanine aminotransferase levels by colorimetric/fluorometric assay kit (Biovision) according to manufacturer’s instructions.

### Western blotting, enzyme assays, immunohistochemistry and immunofluorescence

Proteins were extracted from livers in cold lysis buffer (50 mM Tris-HCl [pH 7.4], 150 mM NaCl, 1% Triton X-100, 1 mM EDTA, and 0.1% SDS) in the presence of protease inhibitors (Sigma-Aldrich). Nuclear and cytoplasmic protein extracts were prepared using NXTRACT kit (Sigma-Aldrich) according to manufacturer’s instructions. Purity of nuclei was assessed by glyceraldehyde 3-phosphate dehydrogenase (GAPDH), citrate synthase (CS), and histone H3 immunoblots. Isolated nuclei were used immediately for the measurements. Total proteins, nuclear and cytoplasmic fractions quantified by the Bradford method were resolved by SDS-PAGE and transferred onto polyvinylidene difluoride (PVDF) membranes. Western blot analyses were performed with antibodies against PDHC-E1α (Abcam ab168379), acetylated histone H4 (acetyl-K5) (Santa Cruz sc-34264), LDH-A (Abcam ab52488), histone H3 (Abcam ab201456), acetylated histone H3 (acetyl-K9) (Abcam ab61231), CS (Abcam ab96600), β-actin (Novus Biologicals NB600-501) and GAPDH (Santa Cruz sc-32233) diluted in 5% milk in TBST and in 1% bovine serum albumin (BSA) in TBST. As secondary antibody, horseradish peroxidase (HRP)-conjugated antibody was used (GE Healthcare). Protein bands were visualized with a chemiluminescence detection system (Pierce). Band intensities were quantified with Quantity One-4.6.7 Basic (1-D Analysis Software) (Biorad Laboratories).

PDHC enzyme activity was measured as described previously^6^ in nuclear and cytoplasmic extracts. Briefly, the assay mixture contained 30 mM Hepes-KOH, 10 mM β-mercaptoethanol, 1 mM CoASH, 0.1 mM NAD (nicotinamide adenine dinucleotide [oxidized form]), 0.3 mM thiamine pyrophosphate, 0.02% Triton, 10 mM MgCl_2_, and 1 mM CaCl_2_. The enzyme reaction was initiated with 20 mM of ^14^C-Na pyruvate (Perkin Elmer) and incubated for 10 min at 37 °C. The reaction was terminated with 250 μl of 1 N HCl on ice and left at 37 °C for 60 min. The ^14^CO_2_ released from the reaction was captured onto filter paper and transferred into scintillation liquid overnight; the amount of ^14^C was counted by a β-counter (Beckman LS6500). LDH activity in nuclear and cytoplasmic fractions was determined by a colorimetric kinetic QuantiChrom™ Lactate Dehydrogenase Kit (Bioassay Systems). Isolated nuclei were deproteinized to measure levels of acetyl-CoA and lactate using the PicoProbe™ Acetyl-CoA Fluorimetric Assay Kit (Biovision) and Lactate Assay Kit (Sigma-Aldrich), respectively. HDAC activity was determined on nuclear fractions by HDAC Assay Kit (Sigma-Aldrich). Mouse liver specimens were fixed in 4% paraformaldehyde for 12 h, stored in 70% ethanol, and embedded into paraffin blocks. For immunohistochemistry (IHC), 5 μm thick sections were rehydrated and permeabilized in PBS with 0.5% Triton (Sigma-Aldrich) for 20 min. Antigen unmasking was performed in 0.01 M citrate buffer in a microwave oven. Next, sections underwent blocking of endogenous peroxidase activity in methanol/1.5% H_2_O_2_ (Sigma-Aldrich) for 30 min and were incubated with blocking solution (3% BSA [Sigma-Aldrich], 5% donkey serum [Millipore], 1.5% horse serum [Vector Laboratories] 20 mM MgCl_2_, 0.3% Triton [Sigma-Aldrich] in PBS) for 1 h. Sections were incubated with primary antibody anti-PDHC-E1α overnight at 4 °C and with universal biotinylated horse anti-mouse/rabbit IgG secondary antibody (Vector Laboratories) for 1 h. Biotin/avidin-HRP signal amplification was achieved using ABC Elite Kit (Vector Laboratories) according to manufacturer’s instructions. 3,3′-diaminobenzidine (Vector Laboratories) was used as peroxidase substrate. Mayer’s hematoxylin (Bio-Optica) was used as counter-staining. Sections were de-hydrated and mounted in VECTASHIELD® (Vector Laboratories). Paraffin-embedded liver sections were stained with hematoxylin and eosin (H&E) for visualization of necrotic areas. At least three sections from each animal were quantified by NIH ImageJ software version 1.46. Immunofluorescence staining of apoptotic cells was performed by terminal deoxynucleotidyl transferase-mediated deoxyuridine triphosphate nick-end labeling (TUNEL) using In Situ Cell Death Detection Kit (Roche Molecular Biochemicals), according to the manufacturer’s instructions. Percentages of TUNEL-positive cells visualized by fluorescence microscopy were quantified by automated counting (ImageJ) on three fields of view selected randomly from each section. The image capture was performed using Leica DM5000 microscope.

For immunofluorescence, 5 μm thick sections were rehydrated and permeabilized in PBS with 0.5% Triton (Sigma-Aldrich) for 20 min. Antigen unmasking was performed in 0.01 M citrate buffer in a microwave oven. Next, background autofluorescence was quenched by bathing slides in 75 mM of NH_4_Cl for 30 min and sections were incubated with blocking solution (3% BSA [Sigma-Aldrich], 5% donkey serum [Millipore], 20 mM MgCl_2_, 0.3% Triton [Sigma-Aldrich] in PBS) for 1 h. Sections were incubated with primary antibody anti-PDHC-E1α or anti-LDH-A overnight at 4 °C. Primary antibodies were detected with corresponding isotype-specific donkey anti-rabbit secondary antibodies conjugated to a fluorochrome (488, Life Technologies). 0.01% 4′,6-diamidine-2′-phenylindole dihydrochloride (DAPI) was added to the secondary antibody. Sections were mounted in Mowiol (Sigma-Aldrich) mounting medium. Image capture was performed using Leica SPE confocal microscope.

### Affinity purification-liquid chromatography tandem-mass spectrometry

Mouse livers were homogenized in cold lysis buffer (50 mM Tris-HCl pH 7.4, 150 mM NaCl, 1% Triton X-100, 1 mM EDTA, and 0.1% SDS) in the presence of protease inhibitors (Sigma-Aldrich) and incubated with anti-acetylated lysine antibody (Abcam ab190479). Samples were incubated with Dynabeads Protein G (Life Technologies) for 2 h at 4 °C to allow antigen binding to the Dynabeads-Ab complex. To elute target antigens, we used 150 mM of ammonium hydroxide before drying samples in a vacuum concentrator. Samples were resuspended in 200 µl of 50 mM ammonium bicarbonate pH 8.3. Tris(2-carboxyethyl)phosphine was added to 5 mM final concentration and left to incubate at 60 °C for 10 min. Samples were cooled to room temperature before adding 15 mM chloroacetamide. They were then left to incubate in the dark at room temperature for further 15 min. One microgram of trypsin (MS grade, Promega) was added to each reaction and incubated end-over-end at 37 °C for 16 h. A further aliquot of 250 ng of trypsin was added and left to incubate end-over-end at 37 °C for 3 h. Samples were dried completely in a vacuum concentrator and resuspended with 20 µl of 0.1% formic acid pH 3. 5 µl per run were injected by an Easy-nLC 1000 UPLC system. For details of affinity purification (AP)-liquid chromatography tandem-mass spectrometry (LC-MS/MS) please see Supplementary Materials and Methods.

### Gene expression analyses

Gene expression analysis was performed on saline and CD95-Ab injected mice (n = 5 per group). Extraction of total RNA was performed according to the manufacturer’s instructions (Qiagen). Total RNA (500 ng) from each sample was prepared using TruSeq RNA sample prep reagents (Illumina) according to manufacturer’s instructions. The amplified fragmented cDNA of ∼200 base pairs in size were sequenced in paired-end mode using the HiSeq2000 (Illumina) with a read length of 2 × 100 base pairs. Reads were aligned and assigned to transcripts and genes from the Human Gencode Release 19 using RSEM version 1.2.19 with standard parameters.^7^ *P* value adjustment for multiple comparisons was done with the false discovery rate of Benjamini-Hochberg.^8^ In this statistical analysis the threshold for statistical significance chosen was a false discovery rate of ≤0.05. For differential expression analysis, an additional threshold was used: logFC ≥2 for upregulated genes and logFC ≤−2 for downregulated genes. For details of gene expression analysis please see Supplementary Materials and Methods.

### Real time PCR

Total RNA from cells and livers was extracted using RNeasy mini kit (Qiagen). One microgram of RNA was retro-transcribed using High-Capacity cDNA Reverse Transcription Kit (Applied Biosystems). The qPCR reactions were set up using SYBR Green Master Mix and run in duplicate on a Light Cycler 480 System (Roche). The primer sequences for mouse *Tnf* were: forward, 5′-ctgaacttcggggtgatcgg-3′; and reverse, 5′-ggcttgtcactcgaattttgaga-3′. The primer sequences for mouse *Il6* were: forward, 5′-ccggagaggagacttcacag-3′; and reverse, 5′-cagaattgccattgcacaac-3′. The primer sequences for mouse *B2m* were: forward, 5′-tggtgcttgtctcactgacc-3′; and reverse, 5′-gtatgttcggcttcccattc-3′. Running program was as follows: pre-heating, 5 min at 95 °C; 40 cycles of 15 s at 95 °C, 15 s at 60 °C, and 25 s at 72 °C. *B2m* was used as housekeeping gene. Data were analyzed by LightCycler 480 software version 1.5 (Roche).

### Cell studies

HeLa and Hepa1-6 cells were cultured in Dulbecco’s modified Eagle’s medium and 10% fetal bovine serum. Routine testing for *Mycoplasma* was not performed. PDHC-E1α-siRNA or LDH-A-siRNA (Ambion) at the final concentrations of 10 nM and 200 nM respectively, or control scrambled siRNA (Sigma-Aldrich) were transfected using INTERFERin® siRNA transfection reagent (Polyplus Transfection). After 48 h of transfection or 24 h of incubation with 750 μM of galloflavin or vehicle (0.075% DMSO), 10 µg/ml of anti-CD95-Ab (clone CH11 from Merck for HeLa and clone Jo2 from BD Biosciences for Hepa1-6) was added to the media. Cells were then collected for Western blot analysis and cell viability by propidium iodide incorporation by flow cytometry. To evaluate cell viability of 10,000 HeLa and Hepa1-6 cells, 1 mM of propidium iodide (Life Technologies V13241) was added in the dark at room temperature for 10 min and the mixture was kept at 4 °C in the dark until analysis. The propidium iodide fluorescence was measured using BD Accuri C6 Plus personal flow cytometer. The forward scatter (FSC) and side scatter (SSC) of particles were simultaneously measured. Cell debris were excluded from analysis by appropriately raising the FSC threshold. The analysis was run in quintuplicate. To evaluate histone acetylation, cells were lysed in cold lysis buffer (50 mM Tris-HCl [pH 7.4], 150 mM NaCl, 1% Triton X-100, 1 mM EDTA, and 0.1% SDS) in the presence of protease inhibitor cocktail (Sigma-Aldrich). Total proteins quantified by the Bradford method were resolved by SDS-PAGE and transferred onto PVDF membrane. Western blot analyses were performed with antibodies against PDHC-E1α (Abcam ab168379), histone H3 (Abcam ab201456), acetylated histone H3 (acetyl-K9) (Abcam ab4441), LDH-A (Abcam ab52488), β-actin (Novus Biologicals NB600-501), and HRP-conjugated secondary antibodies (GE Healthcare) diluted in 5% milk in TBST and 1% BSA in TBST. Protein bands were visualized with a chemiluminescence detection system (Pierce). Band intensities were quantified with Quantity One-4.6.7 Basic (1-D Analysis Software) (Biorad Laboratories).

### Docking studies

Protein-ligand docking simulations were performed using AutoDock Vina tool^9^ and PyRx^10^ for initial screening. The structure was first corrected for errors using the repair tool under the program FoldX.^11^ The initial PDHC models of the octameric or tetrameric structure (chains A-D) as such or after removal of H_2_O, TPP, K^+1^ and Mn^2+^ ions were generated by building hydrogen atoms for the crystal structure of human PDHC (Protein Data Bank [PDB] chain ID 3exe) and by adding Gasteiger charges. Initial conformation of the ligand (galloflavin) was generated by Cartesian optimization of the ligand model in the GROMOS87 force field (PRODRG at: http://davapc1.bioch.dundee.ac.uk/prodrg/submit2.html). All side chains and the backbone of the protein were kept rigid as in the crystal structure. Docking was first performed by placing the ligand in a random position by centering the grid on the macromolecule and setting the grid with a 1-Å spacing on the entire protein; after the identification of the best binding sites, further analysis was performed by starting with the ligand in the binding pockets and setting the grid with a 0.375-Å spacing. The affinity (expressed in kcal/mol) was calculated as the difference in the free energy of binding (ΔG) between the protein and the complex. Control of the docking procedure was obtained by docking galloflavin on the LDH structure (PDB chain ID 1l10). A binding pose was found near the NADH binding site for galloflavin.^12^ This pose with energy ranging between −7.1 and −7.5 ranks third in the list obtained in our protocol. Results were visualized using the Phyton Molecular Viewer program 1.5.6.^13^

### Statistics

Two tailed Student’s *t* test, ANOVA, Tukey’s *post hoc* test, and likelihood ratio test for a generalized linear model were used as statistical tests for mean comparisons. Statistical analyses were performed using R Stats package and MASS. Cumulative survival of mice was assessed by Kaplan-Meier and statistical significance was calculated using long-rank test (GraphPad Prism 7). Experimental group sizes are reported in figure legends. Data are reported as average ± standard deviations.

For further details regarding the materials used, please refer to the CTAT table and supplementary information.

## Results

### Acute liver injuries induce nuclear translocations of PDHC and LDH

**Fig. 1:**
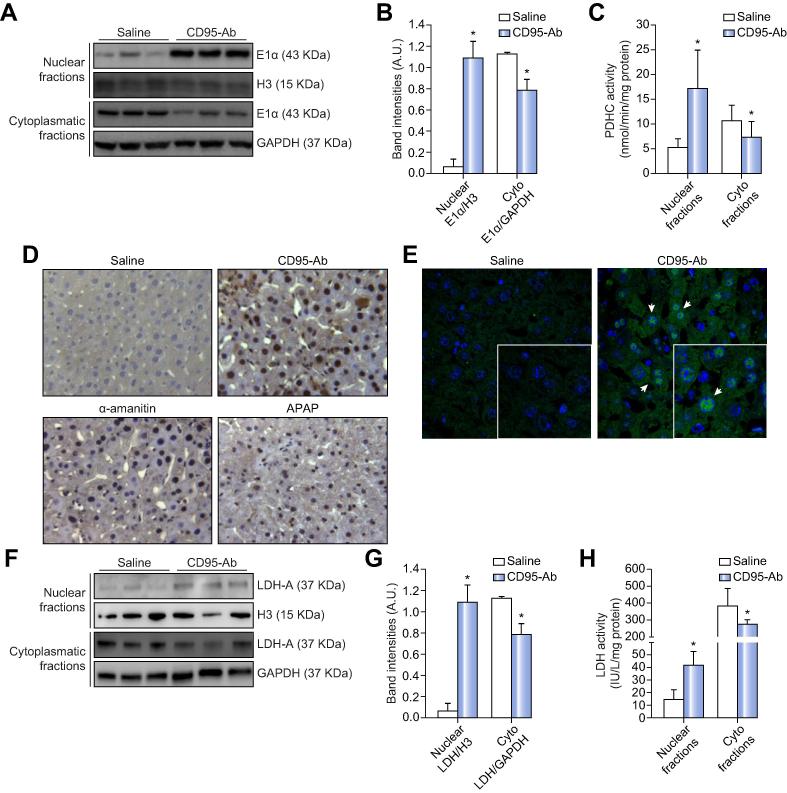
**Acute liver injuries induce nuclear translocations of mitochondrial PDHC and cytosolic LDH.** (A) Western blotting for the E1α subunit of the PDHC on nuclear and cytoplasmic fractions of livers harvested 6 h after the injections of either saline or CD95-Ab. H3 and GAPDH were used as a loading control for the nuclear and cytoplasmic fractions, respectively. (B) Densitometric quantifications of E1α bands in nuclear and cytoplasmic fractions normalized for H3 or GAPDH, respectively (n = 3 mice per group). (C) PDHC activity on nuclear and cytoplasmic fractions of livers harvested 6 h after the injections of either saline (n = 6) or CD95-Ab (n = 9). (D) Representative IHC staining for the E1α subunit of PDHC in livers harvested 6 h after the injections of various hepatotoxins including CD95-Ab, α-amanitin, or APAP. Images were magnified with a ×40 objective. (E) Representative immunofluorescence staining with antibody against the E1α subunit of PDHC in livers harvested 6 h after the injections of CD95-Ab. Nuclei were stained with DAPI. White arrows point to some E1α-positive nuclei. Images were magnified with a ×63 objective. Insets show higher magnification of selected areas. (F) Western blotting for LDH-A on nuclear and cytoplasmic fractions of livers harvested 6 h after injections of either saline or CD95-Ab. H3 and GAPDH were used as loading controls of nuclear or cytoplasmic fractions, respectively. (G) Densitometric quantifications of the LDH-A bands in the nuclear and cytoplasmic fractions normalized for H3 or GAPDH, respectively (n = 3 mice per group). (H) LDH activity in nuclear and cytoplasmic fractions of livers harvested 6 h after injections of either saline (n = 3) or CD95-Ab (n = 6). Means ± SDs are shown; *t* test; *p <0.05. Ab, antibody; A.U., arbitrary units; APAP, acetaminophen; GAPDH, glyceraldehyde 3-phosphate dehydrogenase; IHC, immunohistochemistry; LDH, lactate dehydrogenase; PDHC, pyruvate dehydrogenase complex. (This figure appears in colour on the web.)

Acute liver failure is characterized by a sudden and massive death of liver cells. Hepatocytes are very sensitive to FAS-induced apoptosis and administration of CD95-Ab results in rapid death in mice due to fulminant hemorrhagic hepatitis, mimicking acute liver failure in humans.^14^ α-amanitin and APAP also recapitulate acute liver failure in mice.^15^ Nuclear fractions of livers from mice injected with CD95-Ab showed increased levels of the E1α subunit of PDHC compared to saline-injected controls (Fig. 1A–B). Immunoblot with antibodies against GAPDH and CS confirmed that histone-positive nuclear fractions were free of cytoplasmic and mitochondrial matrix while cytoplasmic fractions were positive for mitochondrial CS (not shown). A concomitant decrease in non-nuclear E1α levels was also observed suggesting translocation of PDHC from the mitochondria to the nuclei (Fig. 1A–B). Compared to saline-injected controls, PDHC activity in CS-free and H3-positive nuclear fractions was increased in mice injected with CD95-Ab (Fig. 1C). Furthermore, increased E1α nuclear signal was detected in mice injected with CD95-Ab compared to saline controls by liver IHC (Fig. 1D) and immunofluorescence (Fig. 1E). A time course study revealed that a greater increase in E1α subunit and PDHC activity in hepatic nuclear fractions occurred at 6 h after CD95-Ab administration (Fig. S1A–B), corresponding to the point of maximal liver damage as shown by serum alanine aminotransferase levels (Fig. S1C), consistent with previous studies.[16], [17]

Increased E1α and PDHC activity were also observed in the livers of mice injected with α-amanitin or APAP by immunoblots and enzyme activity on nuclear fractions, immunofluorescence (Fig. S2A–G), and IHC in the nuclei of hepatocytes (Fig. 1D). In summary, increased nuclear PDHC (nPDHC) was observed in livers of mice with acute liver failure induced by three different hepatotoxic agents.

Increased levels of nuclear LDH (nLDH) were also detected by immunoblots and enzyme activity on nuclear fractions and by immunofluorescence of livers from mice injected with CD95-Ab, α-amanitin, and APAP (Fig. 1F–H, Fig. S3A–D).

### Liver injuries induce intra-nuclear metabolic changes and histone hyper-acetylation

**Fig. 2:**
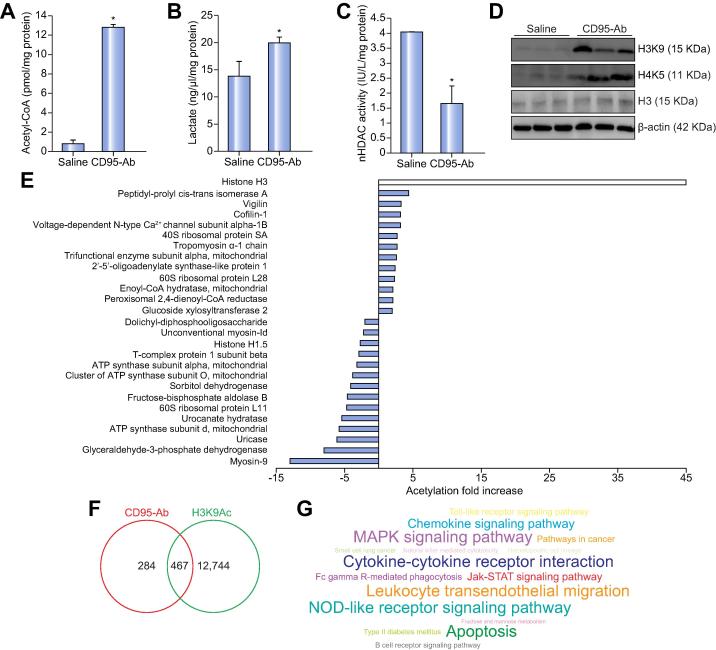
**Liver injury induces intra-nuclear metabolic changes and histone hyper-acetylation.** Nuclear (A) acetyl-CoA, (B) lactate, and (C) nHDAC activity in livers harvested 6 h after the injection of either saline or CD95-Ab (n = 3 per group). (D) Western blotting for acetylated histone H3 (H3K9) and H4 (H4K5) on livers harvested 6 h after the injections of either saline or CD95-Ab. Histone H3 and β-actin were used as loading controls. Each line corresponds to an independent mouse. (E) Affinity purification-liquid chromatography tandem-mass spectrometry of liver proteins enriched by immunoprecipitation with acetyl lysine-specific antibody revealed 13 proteins with fold-change >2 in CD95-Ab *vs.* saline liver samples (n = 5 per group). (F) Venn diagram including the 751 genes upregulated in livers of mice injected with CD95-Ab and the H3K9Ac ChIP-seq data on eight-week-old mouse livers in ENCODE showing a high degree of overlap (Hypergeometric test; *p* value: 9.643501e^−58^). (G) Chart generated using custom annotation scripts shows that response to external stimulus including inflammatory and defense response processes, and cytokine-cytokine receptor interaction pathways were enriched among the 467 genes overlapping between the two groups according to KEGG pathways. Means ± SDs are shown; *t* test; *p <0.05. Ab, antibody; nHDAC, nuclear histone deacetylase. (This figure appears in colour on the web.)

PDHC converts pyruvate into acetyl-CoA, the substrate of lysine acetyltransferases which transfer the acetyl groups to lysines of histones and several other proteins.^18^ Because it is membrane impermeable and very unstable, acetyl-coA has to be synthesized in the subcellular compartment where it is required. Levels of acetyl-CoA were significantly increased in GAPDH/CS-free and H3-positive nuclear fractions of livers of mice injected with CD95-Ab compared to saline-injected controls (Fig. 2A). Increases of acetyl-CoA were also detected in nuclear extracts of livers of mice injected with α-amanitin and APAP (Fig. S4A). Increased lactate was also detected in nuclear fractions of livers of mice injected with CD95-Ab, α-amanitin, and APAP compared to saline controls (Fig. 2B and Fig. S4B). Consistent with a previous report of lactate inhibiting HDAC,^5^ reduced HDAC activity was detected in nuclear fractions of livers of mice injected with CD95-Ab (Fig. 2C).

We reasoned that higher activities of nPDHC and nLDH in injured livers result in increased histone acetylation because of elevated nuclear acetyl-coA and lactate inhibiting HDAC activity. Consistent with this hypothesis, Western blotting with antibodies recognizing histone H3 acetylated at lysines 9 and histone H4 acetylated at lysines 5 revealed marked increase in histone acetylation in livers of mice injected with CD95-Ab compared to saline-injected controls (Fig. 2D). A greater increase in H3 acetylation was detected at 6 h after CD95-Ab administration, consistent with the point at which higher levels of PDHC nuclear translocation were detected (Fig. S1A). α-amanitin and APAP also increased H3 acetylation (Figs. S5 and S6). Acetylation affects a variety of proteins^19^ but an AP-LC-MS/MS analysis of proteins enriched by immunoprecipitation with acetyl lysine-specific antibody revealed only 27 proteins with significant fold-change (fold-change ≤−2 or ≥2, *p* ≤0.05) in CD95-Ab compared to saline liver samples (Fig. 2E). Among these proteins, histone H3 exhibited a 45-fold increase in acetylation (Fig. 2E).

**Fig. 3:**
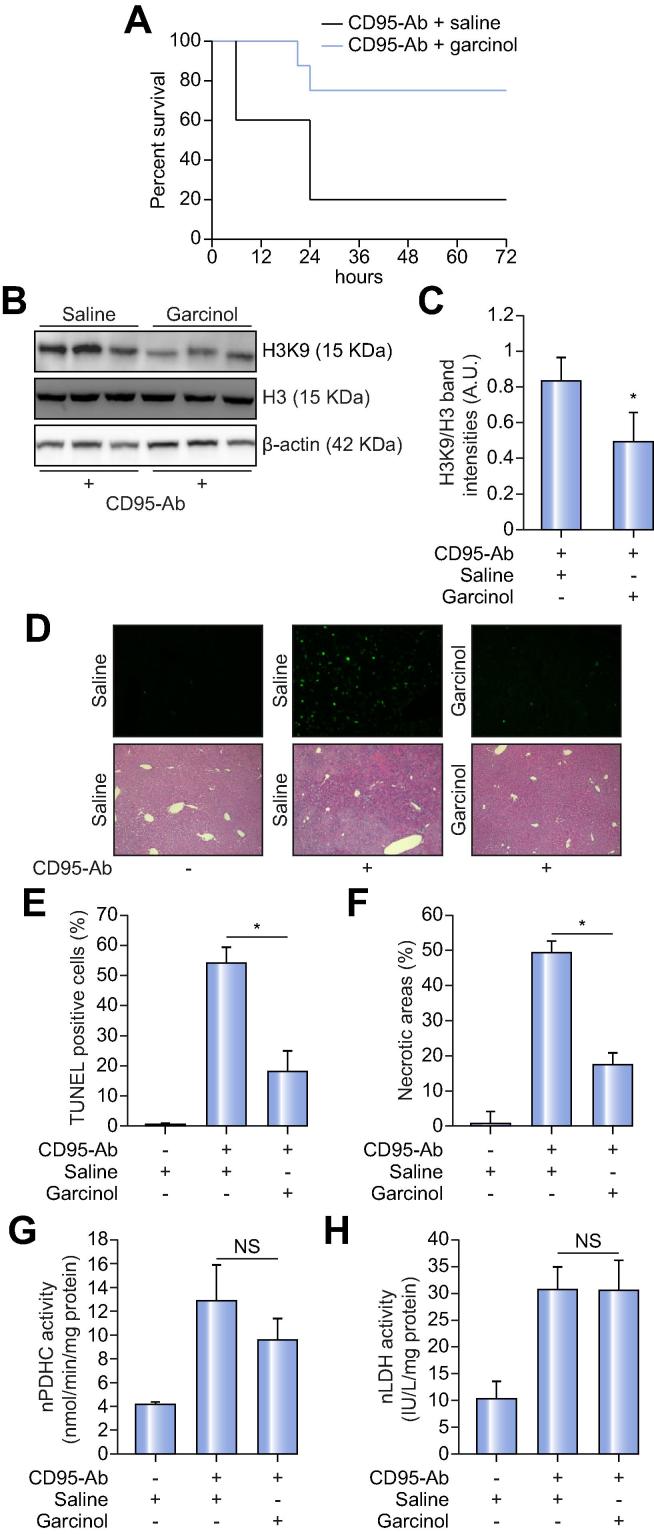
***In vivo* inhibition of histone acetylation by garcinol improves survival and reduces injury in acute liver failure.** (A) Kaplan-Meier survival curves of mice receiving 20 mg/kg/day of garcinol (n = 8) or saline (n = 5) for 5 days prior to the injection of the CD95-Ab. Means ± SDs are shown. Long-rank test *p* = 0.0473. (B) Western blotting for histone H3 (H3) and acetylated histone H3 (H3K9) on livers harvested 6 h after the administrations of CD95-Ab. β-actin was used as a loading control. (C) Densitometric quantifications of H3K9 over H3 from the Western blotting shown in B; *t* test; *p <0.05. (D) Representative TUNEL (upper panels) and H&E (lower panels) staining of livers harvested 6 h after the administrations of CD95-Ab or saline. (E) Quantifications of TUNEL-positive cells and (F) Necrotic areas on livers of at least three sections from each mouse (at least n = 4 mice per group). Means ± SDs are shown. Likelihood ratio test for generalized linear model: *p = 2.2 × 10^−16^. ANOVA plus Tukey’s *post hoc* test; for necrotic area: *p = 2.45 × 10^−8^. Enzyme activities of PDHC (G) and LDH (H) in nuclear fractions (nPDHC and nLDH) of livers harvested 6 h after CD95-Ab injections in mice treated with garcinol (n = 5) or saline (n = 4). Means ± SDs are shown. ANOVA plus Tukey’s *post hoc* test: *p* = 0.11 for nPDHC; *p* = 0.1 for nLDH. Ab, antibody; A.U., arbitrary units; nLDH, nuclear lactate dehydrogenase; nPDHC, nuclear pyruvate dehydrogenase complex; NS, not statistically significant; TUNEL, terminal deoxynucleotidyl transferase dUTP nick end labeling. (This figure appears in colour on the web.)

Histone acetylation plays a major role in gene regulation and acetylated histones are preferentially associated with transcriptionally active chromatin.^20^ Whole-genome gene expression profiling by RNA-seq in CD95-Ab- and in control saline-injected mice showed that CD95-Ab perturbs mouse liver transcriptome in a statistically significant manner: 6,074 genes in total were differentially expressed (GSE 101822). We found 751 genes out of 6,074 were the most significantly induced genes in livers of mice injected with CD95-Ab (Table S1). Comparing the list of 751 of top upregulated genes with the H3K9Ac ChIP-seq data on livers of eight-week old mice in ENCODE,^21^ we found a statistically significant high degree of overlap (467 out of 751; 62%) (Fig. 2F and Table S2). Both gene ontology enrichment analysis (GOEA)[21], [22] (Table S3) and KEGG analyses^23^ (Table S4) performed on these 467 genes revealed that responses to external stimuli, including the inflammatory, defense response processes and cytokine-cytokine receptor interaction pathways were significantly enriched (Fig. 2G). Taken together, these results suggest that increased acetylation of histone H3 results in upregulation of genes related to response to damage that possibly have negative consequences on cell survival. Therefore, we evaluated whether inhibition of histone acetylation by garcinol, a potent inhibitor of histone acetyltransferases p300,^24^ is effective at improving survival in acute liver failure. C57BL/6 mice treated with 20 mg/kg/day garcinol prior to the administration of CD95-Ab showed a significant increase of survival compared to vehicle-injected mice (Fig. 3A). As expected, livers of garcinol-treated mice showed reduced acetylated H3 (Fig. 3B–C). Moreover, garcinol-treated mice showed reduced apoptosis and necrotic liver areas by TUNEL staining and H&E, respectively (Fig. 3D–F). The improved survival and reduced liver damage were not dependent upon reduced nLDH and nPDHC that remained increased in livers of garcinol-treated mice compared to controls (Fig. 3G–H).

### Inhibition of PDH and LDH improves cell viability and survival in acute liver failure

**Fig. 4:**
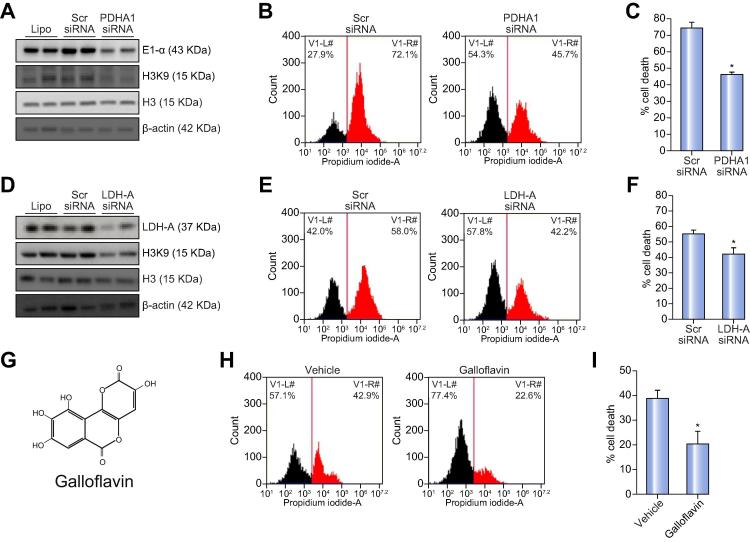
**Inhibition of PDHC and LDH improves cell viability.** (A) Western blotting for the E1α subunit of the PDHC and acetylated histone H3 (H3K9) of HeLa cells transfected with scrambled (scr) siRNA or anti-E1α siRNA (PDHA1 siRNA) before incubations with anti-human CD95-Ab (clone CH11). Histone H3 and β-actin were used as loading controls. (B) Flow cytometry histograms of cells positive (death cells, in red) or negative for propidium iodide (in black) following incubations with CD95-Ab. (C) Averages of the percentages of death cells after incubation with CD95-Ab in cells knock-down for E1α or in control cells transfected with scr siRNA (n = 5 for group). Likelihood ratio for generalized linear model: *p* = 2.2 × 10^−16^. (D) Western blotting for LDH-A and acetylated histone H3 (H3K9) of HeLa cells transfected with scr siRNA or anti-LDH-A siRNA before incubations with anti-human CD95-Ab (clone CH11). Histone H3 and β-actin were used as loading controls. (E) Flow cytometry histograms of cells positive (death cells, in red) or negative (in black) for propidium iodide following incubations with CD95-Ab. (F) Averages of the percentages of death cells after incubation with CD95-Ab in cells knock-down for LDH-A or in control cells transfected with scr siRNA (n = 5 for group). Likelihood ratio for generalized linear model: *p* = 8.9 × 10^−10^. (G) Chemical structure of galloflavin. (H) Flow cytometry histograms of cells positive (death cells, in red) or negative (in black) for propidium iodide following incubations with the CD95-Ab and galloflavin or vehicle (0.075% DMSO). (I) Averages of the percentages of death cells after incubation with CD95-Ab (clone CH11) in cells incubated with galloflavin or vehicle (n = 5 for group). Likelihood ratio for generalized linear model: *p* = 0.001. Ab, antibody; LDH, lactate dehydrogenase; PDHC, pyruvate dehydrogenase complex; scr siRNA, scrambled siRNA. (This figure appears in colour on the web.)

**Fig. 5:**
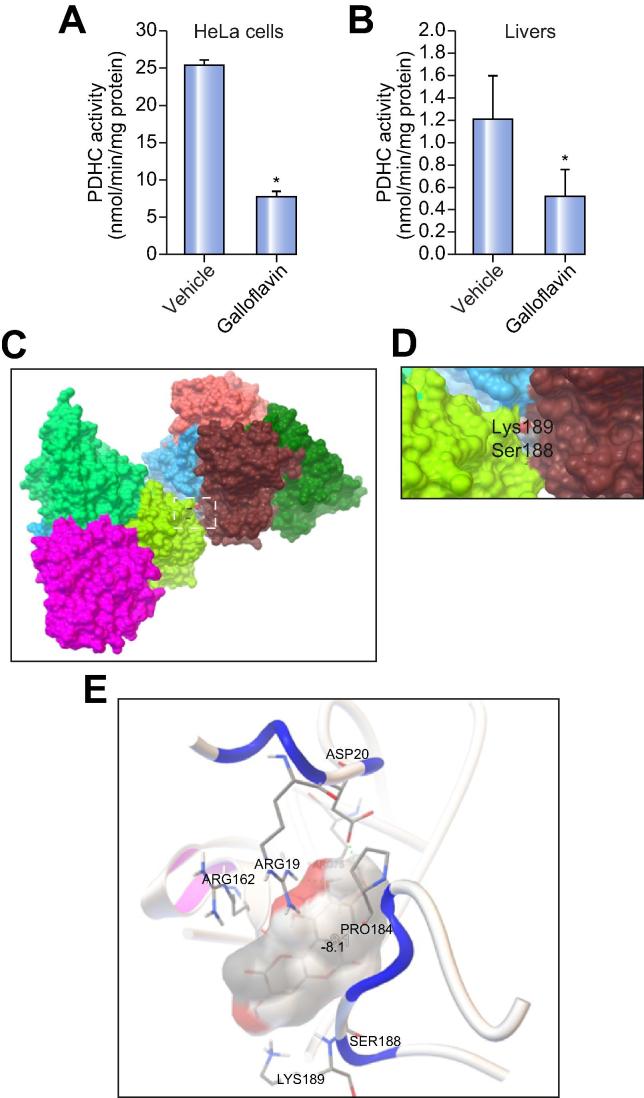
**Galloflavin inhibits PDHC.** (A) PDHC activity in HeLa cells incubated with 750 µM of galloflavin or vehicle (0.075% DMSO) for 6 h (n = 2; *t* test, *p* = 0.011). (B) PDHC activity in mouse livers harvested 6 h after the intraperitoneal injection of a dose of 80 mg/kg of galloflavin compared to vehicle (DMSO 40% in saline) (n = 4 per group; *t* test, *p* = 0.041). (C-D) Surface representation of octameric E1 subunit of the PDHC (3exe) with highlighted point of contact with galloflavin (inset). (E) Three hydrogen bonds were observed between galloflavin and Arg19 and Asp20 of chain D of one tetramer and Lys189 of chain H of the other E1 tetramer. PDHC, pyruvate dehydrogenase complex. (This figure appears in colour on the web.)

Following incubation with pro-apoptotic CD95-Ab, HeLa cells knocked down for PDHA1 or LDH-A showed reduced histone acetylation and improved cell viability (Fig. 4A–F). An improvement of cell viability was also observed in both HeLa and Hepa1-6 cells incubated with galloflavin that inhibits LDH^12^ (Fig. 4G–I, Fig. S7). The greater improvement of cell viability with galloflavin led us to hypothesize that galloflavin is also an inhibitor of PDHC. This hypothesis was supported by inhibition of PDHC activity in HeLa and Hepa1-6 cells following incubation with galloflavin (Fig. 5A and Fig. S8) and in livers of mice injected with 80 mg/kg of galloflavin (Fig. 5B). By docking galloflavin on E1 3D structure of the human PDHC (3exe), a binding site was found between the two tetramers of E1 (Fig. 5C–D). The binding energy of the best pose was −8.1 kcal/mol but other conformations ranging from −7.8 to −7.6 fitted the same binding site. When docked on the tetramer chains A-D no docking was observed. Three hydrogen bonds were observed between galloflavin and the E1 tetramer: two between Arg19 and Asp20 of chain D of one tetramer and Lys189 of chain H of the other tetramer (Fig. 5E). Galloflavin also has contacts with Pro184 and Ser188 on one side and Arg75 and Arg162 on the other side (Fig. S9). One of the four K^+^ ions that stabilize the structure is located near to this site.

**Fig. 6:**
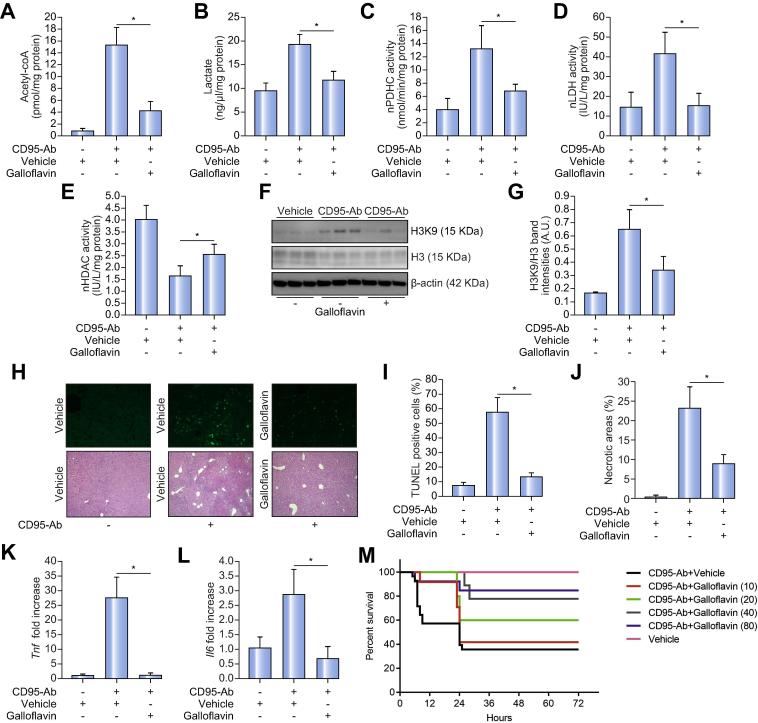
***In vivo* inhibition of PDHC and LDH reduces liver injury and increases survival in acute liver failure.** Concentrations of (A) acetyl-CoA and (B) lactate in nuclear fractions of livers of mice treated with 80 mg/kg/day of galloflavin (n = 4) compared to mice injected with vehicle (DMSO 40% in saline; n = 5). As controls, levels of the metabolites in nuclear fractions of livers of mice receiving only vehicle are shown (n = 4). Means ± SDs are shown. ANOVA plus Tukey’s *post hoc* test: *p = 0.00001 for nuclear acetyl-CoA; *p* = 0.0001 for nuclear lactate. Enzyme activity of (C) PDHC and (D) LDH in nuclear fractions of livers of mice treated with 80 mg/kg/day of galloflavin (n = 4) compared to mice injected with vehicle (n = 5). (E) HDAC activity in nuclear fractions of mice treated with galloflavin (n = 4) compared to controls injected with vehicle (n = 5). As controls, levels of enzyme activities in nuclear fractions of livers of mice receiving only vehicle are shown (n = 5). Means ± SDs are shown. ANOVA plus Tukey’s *post hoc* test: *p* = 0.0211 for nPDHC; *p* = 0.00131 for nLDH; *p* = 0.0416 for nHDAC. (F) Western blotting for histone H3 (H3) and acetylated histone H3 (H3K9) on livers of mice receiving 80 mg/kg/day of galloflavin or vehicle. β-actin was used as a loading control. (G) Densitometric quantifications of H3K9 over H3 from the Western blotting shown in F; *t* test; *p <0.05. (H) Representative TUNEL (upper panels) and H&E (lower panels) staining of livers of mice receiving 80 mg/kg/day galloflavin or vehicle. (I) Quantifications of TUNEL-positive cells and (J) necrotic areas on livers of at least three sections from each mouse (at least n = 4 mice per group). Means ± SDs are shown. Likelihood ratio test for generalized linear model: *p = 1.74 × 10^−5^. ANOVA plus Tukey’s *post hoc* test; for necrotic area: *p = 0.0571853. Expression levels of (K) *Tnf* and (L) *Il6* in livers of mice receiving 80 mg/kg/day galloflavin or vehicle (at least n = 3 per group). Means ± SDs are shown. ANOVA plus Tukey’s *post hoc* test: *p = 0.0000444 for *Tnf* and *p = 0.0026428 for *Il6*. All determinations in panels A to L were performed 6 h after CD95-Ab. (M) Kaplan-Meier survival curves of mice receiving increasing doses of galloflavin [10 mg/kg/day (n = 24; *p* = 0.1865), 20 mg/kg/day (n = 20; *p* = 0.0239), 40 mg/kg/day (n = 13; *p* = 0.0107), and 80 mg/kg/day (n = 10; *p* = 0.0173)] or vehicle. The numbers in parenthesis indicate the dose of galloflavin in mg/kg/day. Long-rank test *p* = 0.0067. Means ± SDs are shown. Ab, antibody; A.U., arbitrary units; nHDAC, nuclear histone deacetylase; nLDH, nuclear lactate dehydrogenase; nPDHC, nuclear pyruvate dehydrogenase complex; n.s, not significant; TUNEL, terminal deoxynucleotidyl transferase dUTP nick end labeling.

Next, we hypothesized that inhibition of nPDHC and nLDH reduces intra-nuclear acetyl-CoA and lactate resulting in reduced histone acetylation and improvement of acute liver failure. To investigate this hypothesis, at the time of CD95-Ab administration we started treatment of C57BL/6 mice with different doses of galloflavin to inhibit nPDHC and nLDH. Both acetyl-coA and lactate were reduced in nuclear fractions of livers of mice injected with galloflavin (Fig. 6A–B). Activities of nPDH and nLDH were both reduced after treatment with galloflavin (Fig. 6C–D) and this reduction was accompanied by increased nuclear HDAC activity (Fig. 6E) and decreased histone H3 acetylation (Fig. 6F–G). Reduced apoptosis and necrotic liver areas by TUNEL staining and H&E, respectively were observed in mice treated with galloflavin compared to vehicle-treated controls (Fig. 6H–J). Moreover, reduced hepatic expression of inflammatory cytokines *Tnf* and *Il6*[25], [26] were detected in galloflavin-injected mice compared to controls (Fig. 6K–L). Importantly, mice injected with galloflavin showed a dose-response increase of survival following CD95-Ab administration compared to saline-injected mice (Fig. 6M). At the higher dose of 80 mg/kg/day survival was 80% in mice treated with galloflavin compared to approximately 40% in vehicle-treated controls (Fig. 6M).

**Fig. 7:**
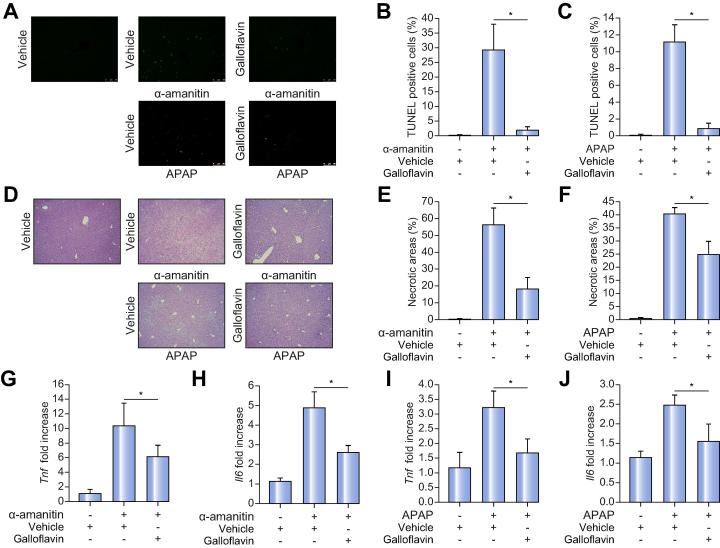
**Galloflavin reduces liver injury induced by α-amanitin or APAP.** Representative (A) TUNEL and (D) H&E staining of livers of mice receiving 80 mg/kg/day galloflavin or saline. Quantifications of (B-C) TUNEL-positive cells and (E-F) necrotic areas on livers of at least three sections from each mouse (at least n = 5 mice per group). Means ± SDs are shown. ANOVA plus Tukey’s *post hoc*; for TUNEL-positive cells: *p = 5.88 × 10^−5^ (α-amanitin) and *p* = 3.25 × 10^−6^ (APAP). For necrotic area: *p = 1.04 × 10^−5^ (α-amanitin) and *p* = 5.57 × 10^−7^ (APAP). Expression levels of (G) *Tnf* and (H) *Il6* in livers of mice receiving 80 mg/kg/day galloflavin or saline from the time of administration of α-amanitin or APAP (at least n = 5 per group). Means ± SDs are shown. ANOVA plus Tukey’s *post hoc* test: *p = 0.024 for *Tnf* and *p = 0.003 for *Il6*. All determinations in panels A to J were performed 6 h after the administration of α-amanitin or APAP. APAP, acetaminophen; TUNEL, terminal deoxynucleotidyl transferase dUTP nick end labeling. (This figure appears in colour on the web.)

Reduced liver apoptosis and necrotic areas, and reduced hepatic expression of *Tnf* and *Il6* were also observed in galloflavin-treated mice with hepatic injury induced by α-amanitin or APAP (Fig. 7A–J).

## Discussion

Translocations of metabolic enzymes to the nucleus link metabolism to gene expression. In this study, we found nuclear translocations of PDHC and LDH in mice with liver injury induced by CD95-Ab, α-amanitin, and APAP. Hepatocytes are very sensitive to Fas-induced apoptosis and CD95-Ab results in rapid death in mice due to fulminant hepatitis, mimicking acute liver failure in humans.^14^ The increased levels of nPDHC and nLDH in livers were associated with higher levels of their products acetyl-CoA and lactate in nuclear fractions, marked increase in histone acetylation and changes in gene expression. Acetyl-CoA is a membrane impermeable and unstable metabolite required for histone acetylation. Increased nuclear lactate is known to have global effects on gene expression via inhibition of HDAC.^5^ Because changes in expression affected genes related to damage response with negative consequences on cell survival, we sought to inhibit nLDH and nPDHC activity in mice with acute liver failure. The reduced liver damage and increased survival of mice treated with the histone acetyltransferase inhibitor garcinol^24^ support the detrimental role of histone hyper-acetylation during acute liver failure.

The two isoforms LDH-A and LDH-B catalyze the same reaction, conversion of pyruvate to lactate at the end of glycolysis. LDH-A is expressed in the liver.^27^ Humans with a hereditary deficiency of the A or B LDH isoforms are free of symptoms, except for muscle rigidity and myoglobinuria following strenuous exercise.[28], [29] Therefore, inhibition of LDH-A by galloflavin is not expected to be toxic. Interestingly, we found that besides LDH inhibition, galloflavin also inhibits PDHC. In contrast to inherited LDH deficiency, PDHC deficiency is responsible for a severe disease in humans.^30^ Nevertheless, inhibition of PDHC by galloflavin does not appear to be complete, although it was sufficient to prevent the increase in activity induced by CD95-Ab. Moreover, toxicity data suggest that galloflavin has good tolerability and doses up to 400 mg/kg injected i.p. that are five times higher than the maximum dose used in this study, did not result in lethal effects in mice.^12^

The mechanism underlying nuclear translocation of metabolic enzymes remains largely unknown. Protein trafficking can be regulated by various proteins that bind and mediate translocation. PDHC translocation appears to involve Hsp70 because its inhibition decreases nuclear PDHC levels.^2^ However, mitochondria-derived vesicles (MDVs) have also been suggested as a mechanism of organelle communication within the cell.^31^ MDVs allow trafficking of mitochondrial proteins to lysosomes.^32^ However, whether they can transfer proteins to the nucleus has not been shown yet. Defining the mechanisms involved in metabolic enzyme nuclear translocation may lead to the development of specific translocation inhibitors with potential applications in acute liver failure.

Acute liver failure is characterized by a sudden and massive death of liver cells. The injured hepatocyte may itself aggravate and exacerbate liver injury by immune activation, often leading to a systemic inflammatory response syndrome that is the most common cause of death. Fas and TNF-α receptor activation are well-characterized processes leading to secretion of TNF-α that aggravates apoptosis^33^, and increased hepatic chemokines that recruit TNF-α-secreting neutrophils to the liver.^34^ Regardless of various etiologies, clinical evidence suggests that acute liver failure is generally associated with significant and uncontrolled activation of systemic inflammation, which may consequently lead to multiple organ failure and poor prognosis.[26], [35] In this study, we found that the pharmacological inhibition of LDH and PDHC by galloflavin reduced liver damage and apoptosis, decreased hepatic cytokine expression and improved survival.

Treatment of acute liver failure is currently based only on supportive measurements and there are no drugs that can change the natural history of the disease. Prior to transplantation most studies reported less than 15% survival in acute liver failure. In the Western world, liver transplantation has improved the chance of survival in patients with acute liver failure. Nevertheless, 20 to 50% of patients listed for liver transplantation die before an organ becomes available as a result of irreversible cerebral edema or sepsis with multi-organ failure.^26^ In contrast, in developing countries the mortality for acute liver failure remains extremely high.^36^ Our results imply that treatment with galloflavin increases resistance to acute liver injury and therefore, it could prolong the time until transplantation or even allow the endogenous regenerative capacity of the liver to rescue the organ. In conclusion, the results of this study suggest that nLDH and/or nPDHC are targets for therapy of patients with acute liver failure of different etiologies.

## Financial support

This work was supported by the European Research Council (IEMTx) and Fondazione Telethon Italy (TCBP37TELC and TCBMT3TELD) to N.B.-P.

## Conflict of interest

The authors declare no conflicts of interest that pertain to this work.

Please refer to the accompanying ICMJE disclosure forms for further details.

## Authors’ contributions

N.B.-P. conceived and designed the research; R.F. performed all the experiments; E.N. helped with the mouse experiments; R.d.C. performed bioinformatic analyses; A.C. performed statistical analyses; G.M. performed docking studies. R.F. and N.B.P. analyzed the data and interpreted the results of the experiments; N.B.-P. wrote the manuscript; R.F., E.N., R.d.C., A.C., G.M. and N.B.-P. approved the final version of the manuscript.
