# Supplementary material for: Pyruvate dehydrogenase complex and lactate dehydrogenase are targets for therapy of acute liver failure
Source: J Hepatol. 2018 Aug;69(2):325–35. doi: 10.1016/j.jhep.2018.03.016 (PMC6057136; doi:10.1016/j.jhep.2018.03.016)
Supplement: Supplementary data 3 [file CTAT_table.pdf]

EASL | JOURNAL OF HEPATOLOGY

Journal of Hepatology

CTAT methods

1.1 Antibodies

Name	Supplier	Cat no.	Clone no.
Histone H3 (acetyl-K9)	Abcam	ab61231	
Ac-histone H4 (Lys 5)	Santa Cruz Biotechnology	sc-34264	
Citrate synthetase	Abcam	ab96600	
Glyceraldeide-3-phosphate dehydrogenase	Santa Cruz Biotechnology	sc-32233	6c5
Lactate Dehydrogenase A	Abcam	ab52488	EP1566Y
Histone H3	Abcam	ab201456	EPR17785
Pyruvate Dehydrogenase E1-α subunit	Abcam	ab168379	EPR11098
B-actin	Novus Biologicals	NB600-501	AC-15
Acetylated Lysine	Abcam	ab190479	RM101
ECL Anti-Mouse IgG, Horseradish Peroxidase-Linked Species-Specific Whole Antibody (from sheep)	GE Healthcare	NA931	
ECL Anti-rabbit IgG, Horseradish Peroxidase-Linked Species-Specific Whole Antibody (from donkey)	GE Healthcare	NA934	
Donkey anti-Rabbit IgG (H+L) Highly Cross-Adsorbed Secondary Antibody, Alexa Fluor 488	Thermo Fisher Scientific	A-21206	

1.2 Cell lines

Name	Supplier	Cat no.	Passage no.	Authentication test method
Hepa1-6	Available in the laboratory	Not applicable	Not recorded	Morphology check by microscope
HeLa	Available in the laboratory	Not applicable	Not recorded	Morphology check by microscope

1.3 Organisms

Name	Supplier	Strain	Sex	Age
C57BL/6 mouse	Charles River	C57BL/6N	Male	6 weeks

1.4 Sequence based reagents: Primers

Name	Sequence	Supplier
Tnf-α forward	5′-ctgaacttcggggtgatcgg-3′	Sigma-Aldrich

Tnf-α reverse	5′-ggcttgtcactcgaattttgaga-3′.	Sigma-Aldrich
Il-6 forward	5′-ccggagaggagacttcacag-3′;	Sigma-Aldrich
Il-6 reverse	5′-cagaattgccattgcacaac-3′.	Sigma-Aldrich
β2-microglobulin forward	5′-tggtgcttgtctcactgacc-3′	Sigma-Aldrich
β2-microglobulin reverse	5′-gtatgttcggcttcccattc-3′.	Sigma-Aldrich
Pre-designed siRNA anti-PDHA1	Sense: ccagggccagauauucgaatt Antisense: uucgaauaucuggcccuggtt	Ambion by Life technologies

1.5 Deposited data

Name of repository	Identifier	Link
GSE 101822	Whole-genome gene expression profiling by RNA-seq in CD95-Ab- and in control saline-injected mice	https://www.ncbi.nlm.nih.gov/geo/query/acc.cgi

1.6 Software

Software name	Manufacturer	Version
Quantity One 1-D Analysis Software	Biorad Laboratories	Version 4.6.7
ImageJ software	NIH Image	Version 1.46.
SequestHT	Thermo Fisher Scientific	Version 1.4.1.14
Proteome Discoverer	Thermo Fisher Scientific	Version 1.4
X!Tandem	The GPM, thegpm.org	Version CYCLONE (2010.12.01.1)
Scaffold	Proteome Software Inc.	Version 4.4.8
RSEM	BMC Bioinformatics	Version 1.2.19
DAVID online tool	Laboratory of Human Retrovirology and Immunoinformatics (LHRI)	Version 6.7
LightCycler 480 software	Roche	Version 1.5
AutoDock Vina tool	The Scripps Research Institute	Version 1.1.2
PyRx	The Scripps Research Institute	Version 0.8
PRODRG	GlycoBioChem.	Version 2
Phyton Molecular Viewer	The Scripps Research Institute	Version 1.5.6
GraphPad Prism	GraphPad Software	Version 7
R packages "stats" and "MASS	CRAN	
ENCODE (Encyclopedia of DNA Elements)	National Human Genome Research Institute (NHGRI)	

1.7 Other (e.g. drugs, proteins, vectors etc.)

Purified NA/LE Hamster Anti-Mouse CD95, clone Jo2	BD Pharmigen	554254
Anti-Fas (human, activating), clone CH11	Merckmillipore	05-201
α-amanitin from Amanita phalloides	Sigma-Aldrich	A2263
Acetaminophen	Sigma-Aldrich	A7085
Garcinol	Enzo Life Science	BML-GR343-0050
Galloflavin	Provided by Dr. Filippo Minutolo	
