# Supplementary material for: Pyruvate dehydrogenase complex and lactate dehydrogenase are targets for therapy of acute liver failure
Source: J Hepatol. 2018 Aug;69(2):325–35. doi: 10.1016/j.jhep.2018.03.016 (PMC6057136; doi:10.1016/j.jhep.2018.03.016)
Supplement: Supplementary data 1 [file mmc1.pdf]

Pyruvate dehydrogenase complex and lactate dehydrogenase are targets for therapy of acute liver failure

Rosa Ferriero, Edoardo Nusco, Rossella De Cegli, Annamaria Carissimo, Giuseppe Manco, Nicola Brunetti-Pierri

Table of contents

Fig. S1 2

Fig. S2 3

Fig. S3 5

Fig. S4 6

Fig. S5 7

Fig. S6 8

Fig. S7 9

Fig. S8 10

Fig. S9 11

Supplementary Materials and Methods 12

Supplementary Figures

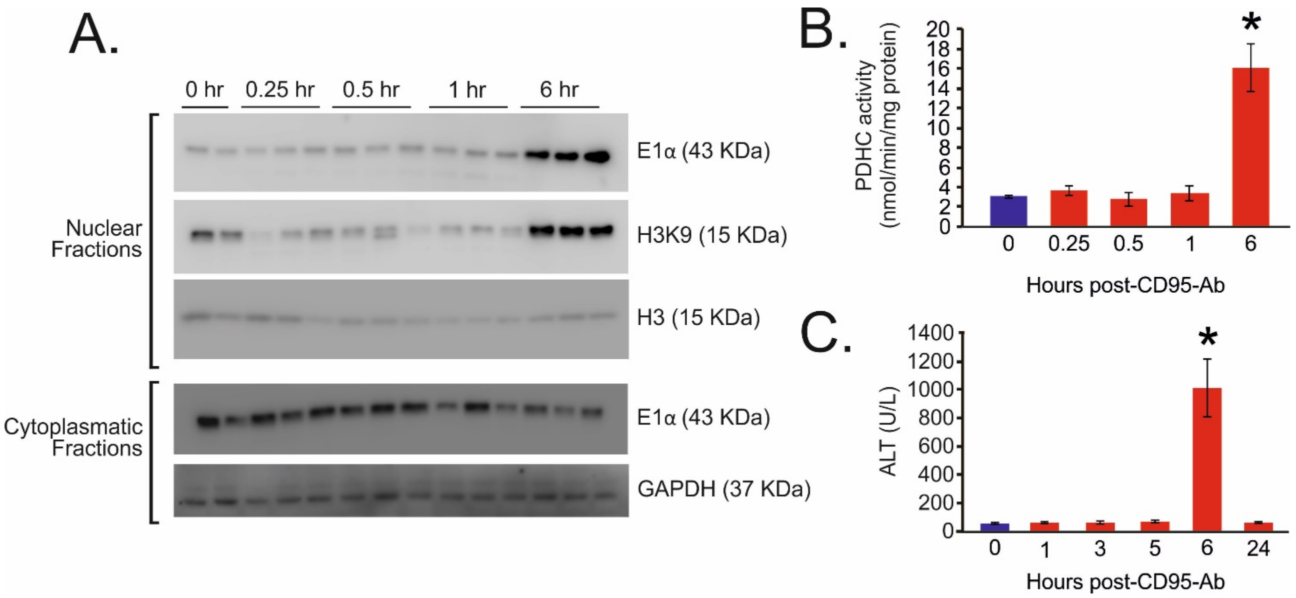

Fig. S1. Nuclear translocations of pyruvate dehydrogenase complex (PDHC) at various times after acute liver injury induced by CD95-Ab.

(A) Western blotting for the E1α subunit of the PDHC on nuclear and cytoplasmic fractions of livers harvested at multiple time points after the injection of CD95-Ab. H3 and GAPDH were used as a loading controls of nuclear and cytoplasmatic fractions, respectively. Each lane corresponds to an independent mouse. **(B)** PDHC activity on liver nuclear fractions of mice at multiple times after the injections of CD95-Ab (n=3 for each time point). ANOVA plus Tukey's *post-hoc*: *p = 0.0010053. (**C**) Serum ALT at multiple times after the injections of CD95-Ab (at least n=3 for each time point). ANOVA plus Tukey's *post-hoc*: *p = 0.0001. Means ± SDs are shown.

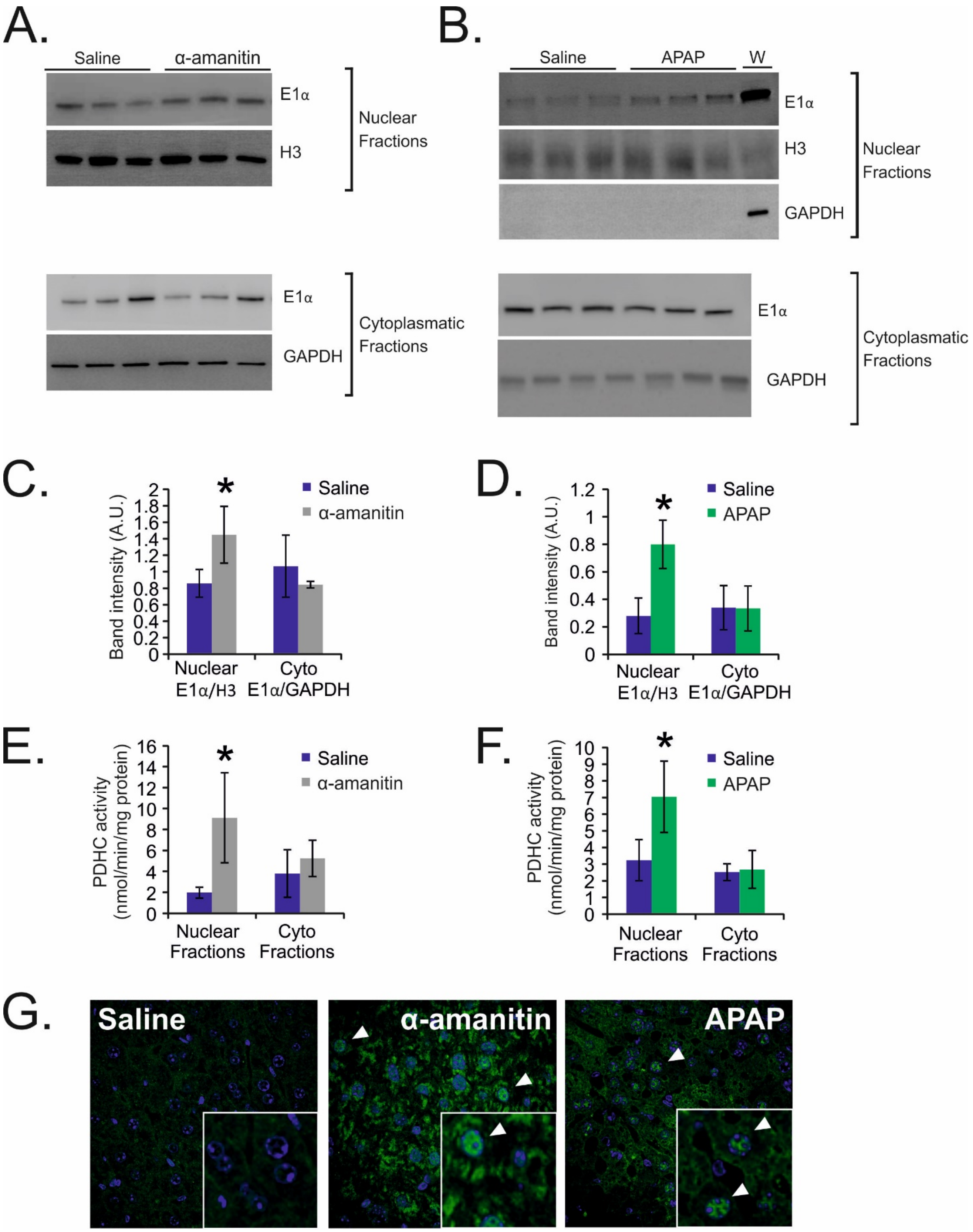
A.
Saline
α-amanitin
E1α
H3
Nuclear Fractions
E1α
GAPDH
Cytoplasmatic Fractions
B.
Saline
APAP
W
E1α
H3
GAPDH
Nuclear Fractions
E1α
GAPDH
Cytoplasmatic Fractions
C.
Band intensity (A.U.)
Saline
α-amanitin
Nuclear E1α/H3
Cyto E1α/GAPDH
D.
Band intensity (A.U.)
Saline
APAP
Nuclear E1α/H3
Cyto E1α/GAPDH
E.
PDHC activity (nmol/min/mg protein)
Saline
α-amanitin
Nuclear Fractions
Cyto Fractions
F.
PDHC activity (nmol/min/mg protein)
Saline
APAP
Nuclear Fractions
Cyto Fractions
G.
Saline
α-amanitin
APAP

Fig. S2. α-amanitin and acetaminophen (APAP) induce nuclear translocations of mitochondrial PDHC.

Western blotting for the E1α subunit of the PDHC on nuclear and cytoplasmic (cyto) fractions of livers harvested 6 hours after the injections of saline, α-amanitin **(A)**, or APAP **(B)**. H3 and GAPDH were used as loading controls of nuclear and cytoplasmatic fractions, respectively. Each lane corresponds to an independent mouse. **(C-D)** Densitometric quantifications of the E1α bands in the nuclear and cytoplasmatic fractions normalized for H3 or GAPDH, respectively (n=3 per group). **(E)** PDHC activity on nuclear and cytoplasmic fractions of livers harvested 6 hours after the injections of saline (n=3) or α-amanitin (n=5). **(F)** PDHC activity on nuclear and cytoplasmic fractions of livers harvested 6 hours after the injections of saline (n=3) or APAP (n=5). **(G)** Representative immunofluorescence stainings with antibody against the E1α subunit of PDHC of livers harvested 6 hours after the injections of saline, α-amanitin, or APAP. Nuclei were stained with DAPI. White arrows points to nuclei positive for E1α. Images were magnified with a ×63 objective. Insets show higher magnification of selected areas. Means ± SDs are shown; *t* test; *$p<0.05$. W= whole extract.

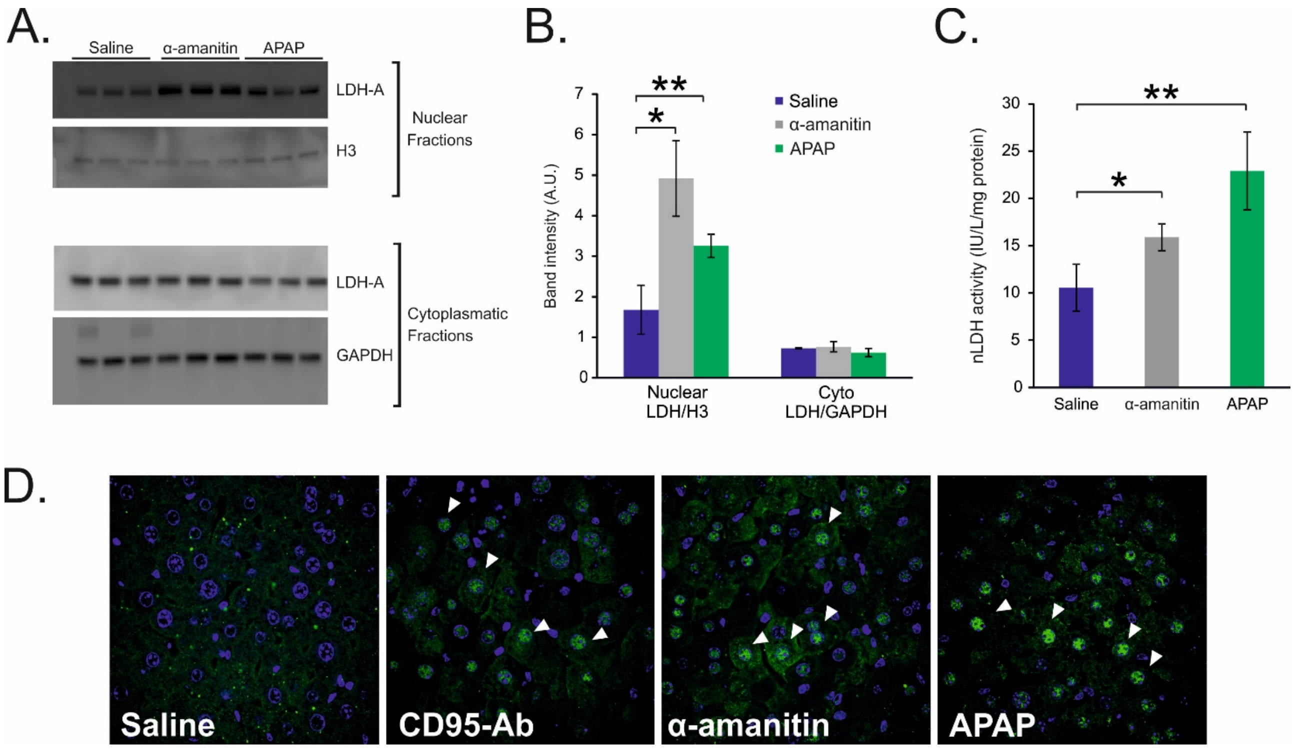

Fig. S3. α-amanitin and acetaminophen (APAP) induce nuclear translocations of lactate dehydrogenase (LDH).

(A) Western blotting for LDH-A on nuclear and cytoplasmic (cyto) fractions of livers harvested 6 hours after the injections of saline, α-amanitin, or APAP. H3 and GAPDH were used as loading controls of nuclear and cytoplasmatic fractions, respectively. Each lane corresponds to an independent mouse. **(B)** Densitometric quantifications of LDH-A bands in nuclear and cytoplasmatic fractions normalized for H3 or GAPDH, respectively (n=3 mice per group). Means ± SDs are shown; ANOVA plus Tukey's *post-hoc*: *p = 0.0015; **p = 0.0427. **(C)** LDH activity in nuclear fractions (nLDH) of livers harvested 6 hours after the injections of saline, α-amanitin, or APAP (n=3 per group). Means ± SDs are shown; ANOVA plus Tukey's *post-hoc*: *p = 0.05; **p = 0.0017. **(D)** Representative immunofluorescence stainings with antibody against LDH-A in livers harvested 6 hours after the injections of saline, CD95-Ab, α-amanitin, or APAP. Nuclei were stained with DAPI. White arrows points to nuclei positive for LDH-A. Images were magnified with a ×63 objective.

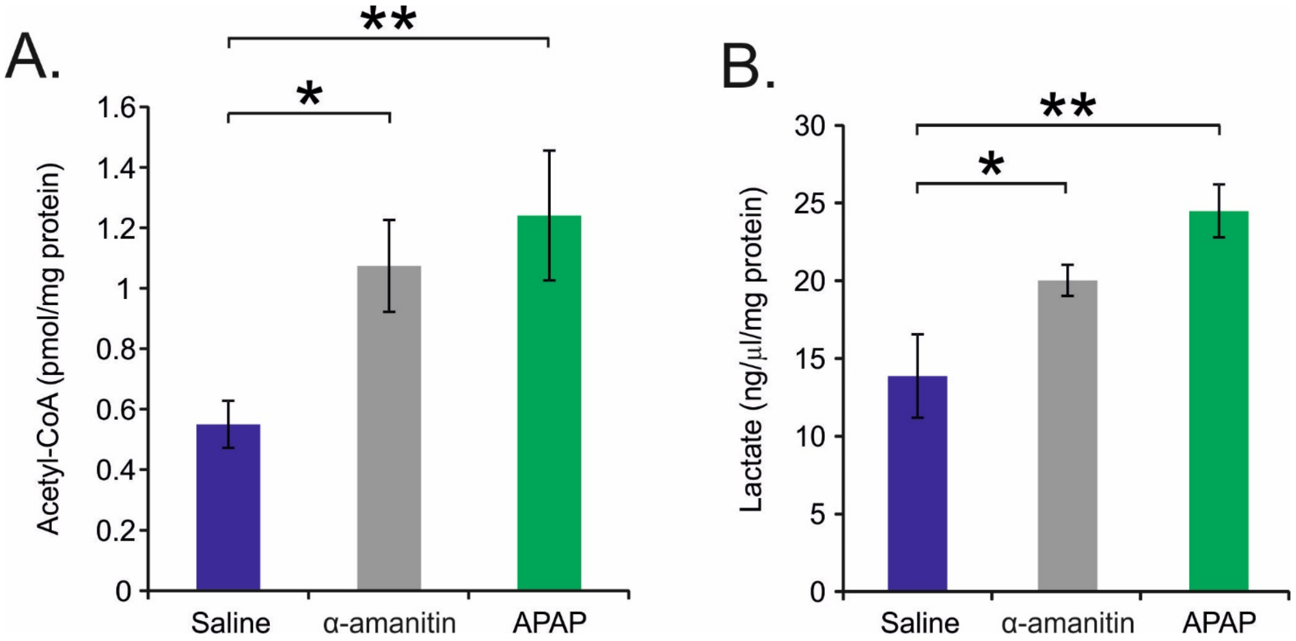

Fig. S4. α-amanitin and APAP induce intra-nuclear metabolite changes.

(**A**) Acetyl-CoA in nuclear fractions of livers harvested 6 hours after the injections of saline (n=4), α-amanitin (n=5), or APAP (n=5). Means ± SDs are shown; ANOVA plus Tukey's *post-hoc*: *p = 0.0137; **p = 0.05. (**B**) Lactate in nuclear fractions of livers harvested 6 hours after the injections of saline, α-amanitin, or APAP (n=3 per group). Means ± SDs are shown; ANOVA plus Tukey's *post-hoc*: *p = 0.0184 **p = 0.0012.

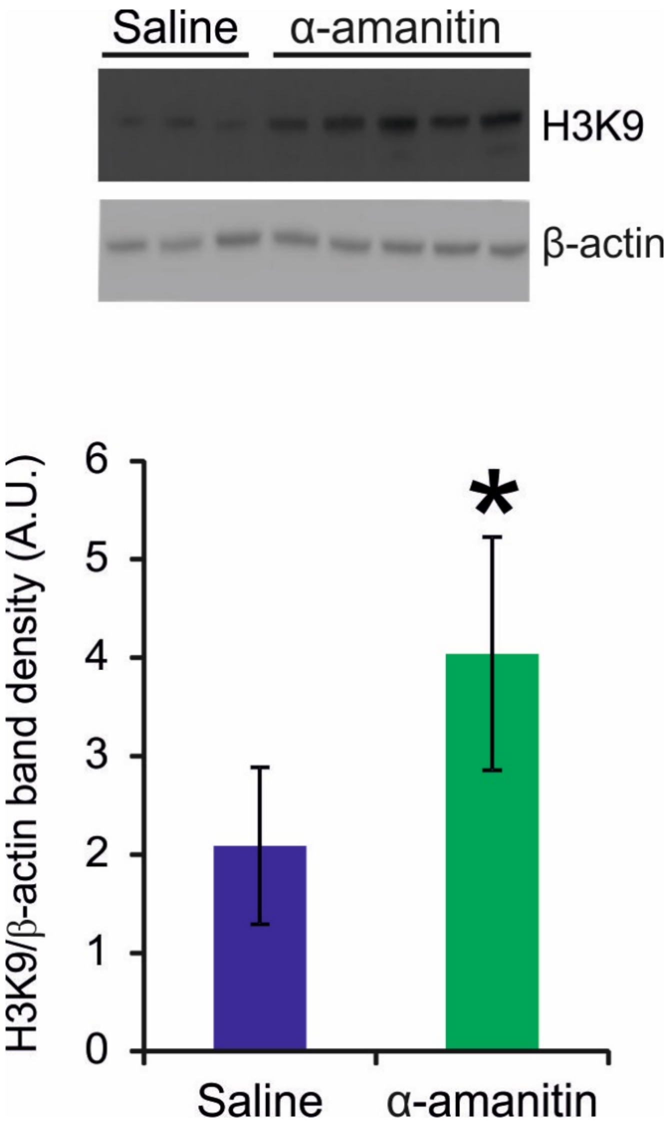

Fig. S5. α-amanitin induces histone hyper-acetylation in livers.

Western blotting for acetylated histone H3 (H3K9) on livers harvested 6 hours after the injections of saline or α-amanitin. β-actin was used as loading control. Each lane corresponds to an independent mouse. The graph shows the densitometric quantifications of acetylated histone H3 (H3K9) bands in liver extracts normalized for β-actin (n=3 mice for saline group and n=5 mice for α-amanitin group). Means ± SDs are shown; *t* test; *$p < 0.05$.

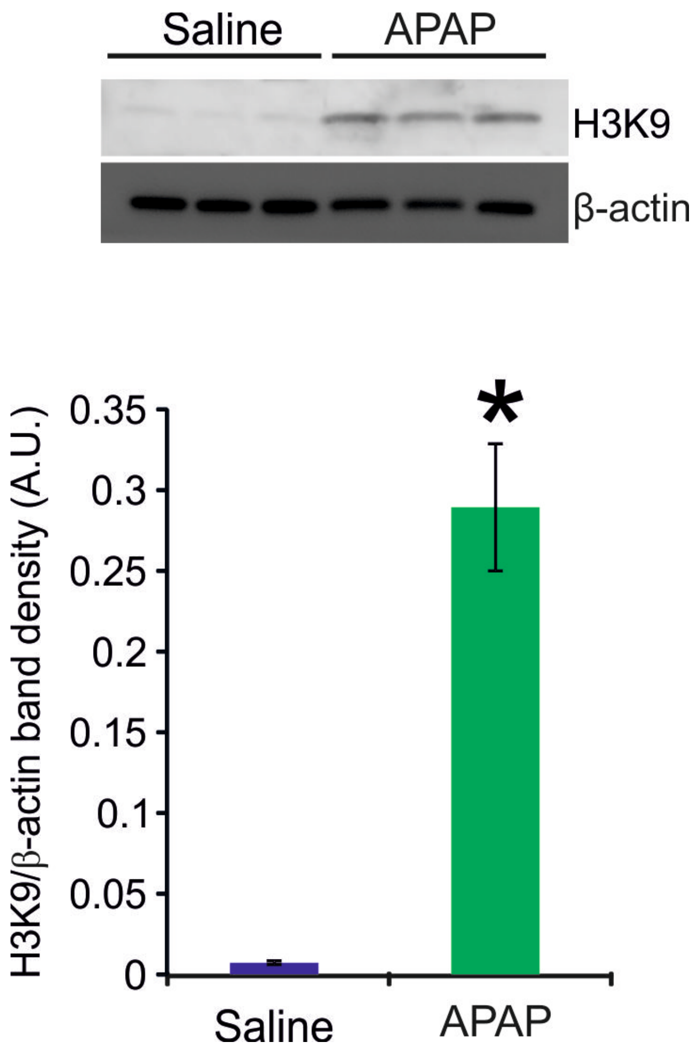

Fig. S6. APAP induces histone hyper-acetylation in livers.

Western blotting for acetylated histone H3 (H3K9) on livers harvested 6 hours after the injections of saline or APAP. β-actin was used as loading control. Each lane corresponds to an independent mouse. The graph shows the densitometric quantifications of acetylated histone H3 (H3K9) bands in liver extracts normalized for β-actin (n=3 mice). Means ± SDs are shown; *t* test; *p < 0.05.

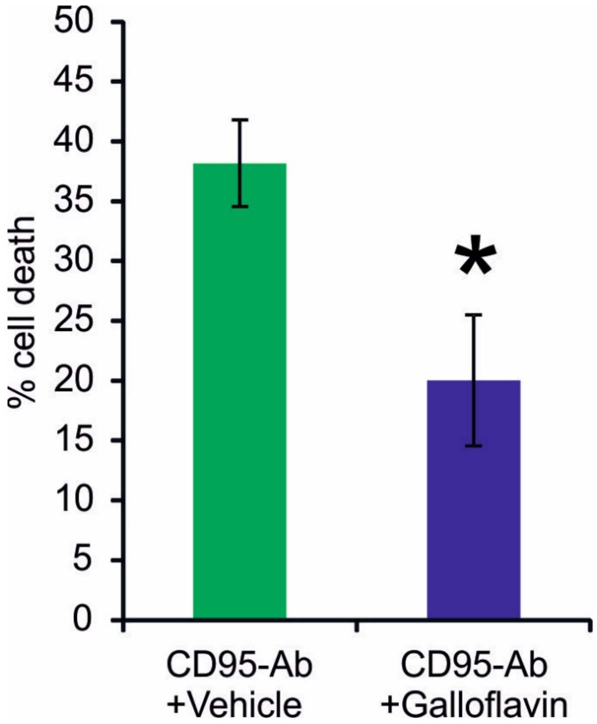

Fig. S7. Galloflavin improves viability of Hepa1,6 cells incubated with CD95-Ab.

Averages of the percentages of death cells evaluated at 6 hours after the incubation with CD95-Ab (clone Jo2) in Hepa1,6 cells treated with vehicle (0.025% DMSO in medium) or galloflavin (n=4 for group). Means ± SDs are shown; likelihood ratio test for generalized linear model: *p = 0.0000002664.

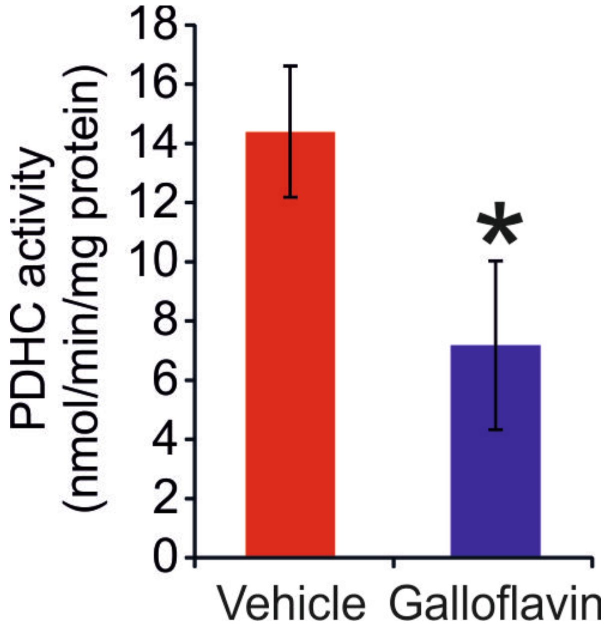

Fig. S8. Galloflavin reduces PDHC activity in Hepa1,6 cells. PDHC activity in Hepa1,6 cells incubated with 750 µM of galloflavin or vehicle (0.025% DMSO in medium) for 6 hours (n= 2 per group). Means ± SDs are shown. *t* test; *p < 0.05.

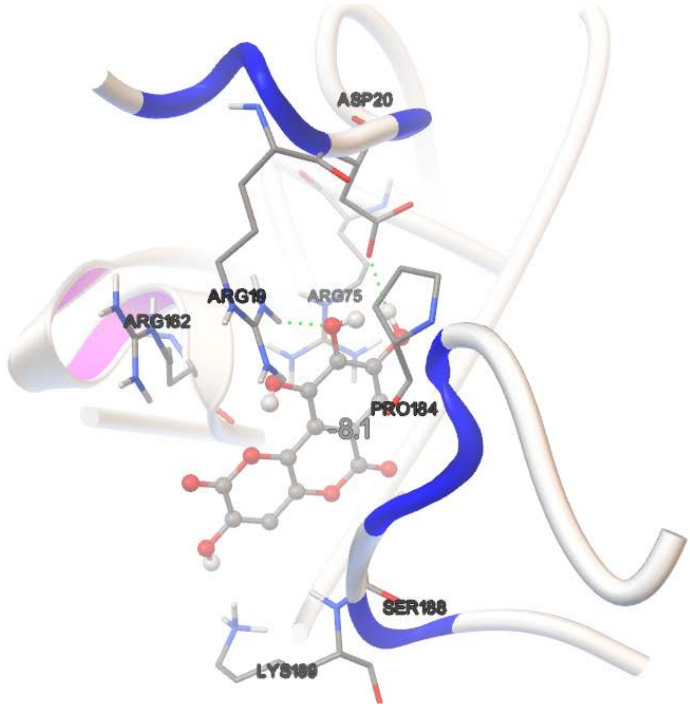

Fig. S9. Details of interactions at the best galloflavin pose (energy -8.1) with the E1 tetramer model (3exe) after removal of H_2O, K^{+1}, Mn^{2+} and TPP.

Supplementary Materials and Methods

***Affinity purification (AP)*-Liquid chromatography tandem-mass spectrometry (LC-MS/MS)**

Samples were loaded directly onto a 45cm long 75µm inner diameter nanocapillary column packed with 1.9µm C18-AQ (Dr. Maisch) mated to PicoTip emitter (New Objective) in-line with Thermo Orbitrap Elite. The mass spectrometer was operated in data dependent mode with the 120,000 resolution MS1 scan (400-1800 m/z) in the Orbitrap followed by up to 20 MS/MS scans in the ion trap. Dynamic exclusion list was invoked to exclude previously sequenced peptides for 120s if sequenced within the last 30s. All MS/MS samples were analyzed using SequestHT (Thermo Fisher Scientific, version 1.4.1.14) within Proteome Discoverer 1.4 suite and X!Tandem [The GPM, thegpm.org; version CYCLONE (2010.12.01.1)] within Scaffold. Sequest and X!Tandem were set up to search Uniprot mouse database (downloaded 19aug14, 45475 entries) assuming the digestion enzyme trypsin. Fragment ion mass tolerance of 0.40 Da and a parent ion tolerance of 10.0 PPM were used. Acetyl-lysine were specified in Sequest and in X!Tandem as variable modifications. Scaffold (version Scaffold_4.4.8, Proteome Software Inc.) was used to validate MS/MS based peptide and protein identifications. Peptide identifications were accepted if they could be established at greater than 97.0% probability to achieve a False Discovery Rate (FDR) less than 1.0%. Peptide Probabilities from Sequest were assigned by the Scaffold Local FDR algorithm. Peptide Probabilities from X!Tandem were assigned by the Peptide Prophet algorithm[1] with Scaffold delta-mass correction. Protein identifications were accepted if they could be established at greater than 5.0% probability to achieve an FDR less than 1.0% and contained at least 2 identified peptides. Protein probabilities were assigned by the Protein Prophet algorithm.[2] Proteins that contained similar peptides and could not be differentiated based on MS/MS analysis alone were grouped to satisfy the principles of parsimony. Proteins sharing significant peptide evidence were grouped into clusters.

Gene expression analyses

The list of 751 induced genes considering the logFC≥2 was crossed with the list of peaks

obtained by the Experiment ENCSR000CEQ publicly available in the Encyclopedia of DNA Elements (ENCODE) project.[3] The list of peaks was cut to restrict the promoter region to a 3kb region which includes the peaks located 2.5 kb upstream and 0.5 kb downstream the transcription start site (TSS). In this region we found 23393 peaks (corresponding to 13211 unique genes) for the H3K9Ac mark enrichment. The overlap with the list of 751 induced genes was high: 62% (467/751) of the induced genes (**Table S2**) presents at least one H3K9Ac positive peak in the mouse liver. To evaluate the significance of the overlap, we applied a hypergeometric test. GOEA[4, 5] was performed on the list of 467 genes resulting from the overlap described above using the DAVID online tool (DAVID Bioinformatics Resources 6.7) restricting the output to all Biological Process terms (BP_ALL) (**Table S3**). KEGG pathway analysis was also performed on this list of genes (**Table S4**). The significance threshold of FDR≤10% and Enrichment Score ≥1.5 were used for the GOEA while FDR≤10% was used in the KEGG pathway analysis. Venn diagram was generated using custom annotation script.

References

1. Keller A, Nesvizhskii AI, Kolker E and Aebersold R. Empirical statistical model to estimate the accuracy of peptide identifications made by MS/MS and database search. Anal Chem 2002;74:5383-92.
2. Nesvizhskii AI, Keller A, Kolker E and Aebersold R. A statistical model for identifying proteins by tandem mass spectrometry. Anal Chem 2003;75:4646-58.
3. Consortium EP. An integrated encyclopedia of DNA elements in the human genome. Nature 2012;489:57-74.
4. Dennis G, Jr., Sherman BT, Hosack DA, Yang J, Gao W, Lane HC, et al. DAVID: Database for Annotation, Visualization, and Integrated Discovery. Genome Biol 2003;4:P3.
5. Huang da W, Sherman BT, Lempicki RA. Systematic and integrative analysis of large gene lists using DAVID bioinformatics resources. Nat Protoc 2009;4:44-57.
